# Cre-ativity in the liver: Transgenic approaches to targeting hepatic nonparenchymal cells

**DOI:** 10.1002/hep.27606

**Published:** 2015-04-13

**Authors:** Stephen N Greenhalgh, Kylie P Conroy, Neil C Henderson

**Affiliations:** 1 MRC Center for Inflammation Research, The Queen’s Medical Research Institute, University of EdinburghEdinburgh, UK

## Abstract

Rapid evolution in transgenic (Tg) mouse technology now permits cell-specific and temporal control of fluorescent cell-labeling and gene inactivation. Here, we discuss the principal strategies that have been utilized to target, label, and manipulate hepatic nonparenchymal cells, with emphasis on the utility of constitutive and inducible Cre-lox systems. We summarize key findings of studies employing Tg technology to target hepatic stellate cells, myofibroblasts, liver sinusoidal endothelial cells, and macrophages to illustrate the power of these approaches in identifying cell-specific molecular mechanisms critical to the pathophysiology of liver disease. Increasing adoption of Tg techniques will help to answer fundamental questions regarding the pathogenesis of hepatic diseases and provide the mechanistic rationale to allow identification of novel drug targets, ultimately translating into effective therapies for patients with liver disease. (Hepatology 2015;61:2091–2099)

Transgenic (Tg) mouse technology has evolved rapidly since the turn of the century, facilitating major advances in our understanding of hepatic pathobiology in murine models of human liver disease. Initial Tg manipulations were restricted to global gene knockout, greatly limiting the study of genes with vital functions during development owing to embryonic or perinatal lethality. These limitations have largely been circumvented through development of techniques, such as Cre-lox, that permit targeted genetic manipulation, allowing both spatial and temporal control of gene expression in mice. Conditional deletion of targeted genomic DNA sequences has become a standard approach, facilitating both interrogation of cell-specific gene function and detailed study of cell ontogeny through fate mapping. Within the liver, these techniques have revolutionized our ability to interrogate the roles of specific genes and cell lineages in a broad range of hepatic disease processes. Here, we discuss how this burgeoning field is being harnessed to explore the myriad roles of hepatic nonparenchymal cells (NPCs) in the pathophysiology of liver disease.

## Tg Systems

### Cre-lox

**Figure 1:**
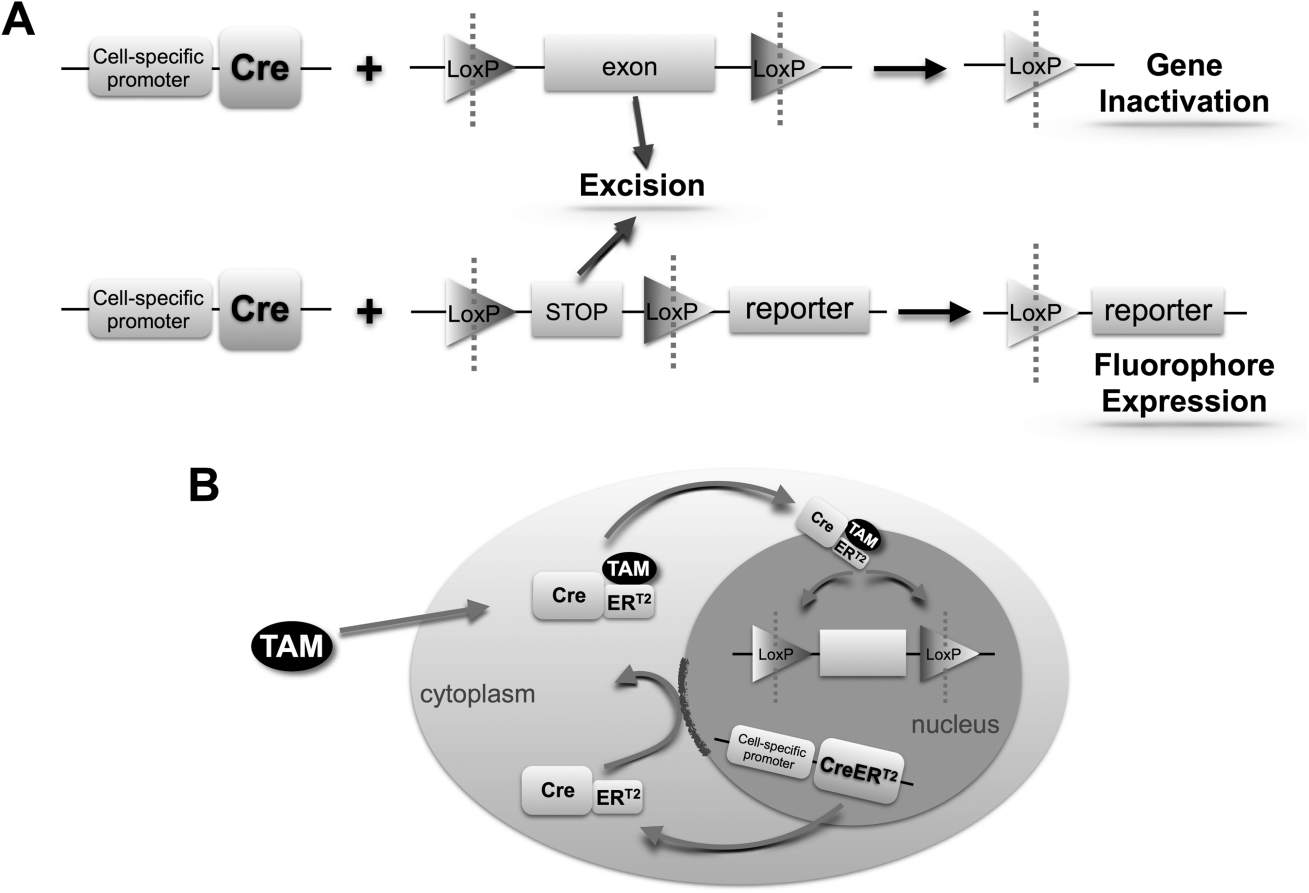
Schematic diagram of cell-specific Cre recombinase-induced gene inactivation and fluorescent reporter expression systems. (A) Cell-specific expression of Cre recombinase results in excision of *loxP*-flanked sequences leading to gene inactivation or fluorescent reporter expression. (B) Spatiotemporal regulation of Cre activity: tamoxifen (TAM) administration permits entry of the Cre-ER^T2^ complex into the nucleus, allowing excision of *loxP*-flanked sequences.

Developed in the 1980s, the Cre-lox system is currently one of the most widely used techniques for genetic manipulation in mice.^1^ The bacteriophage P1 *cre* (cyclization recombinase) gene catalyzes DNA recombination between pairs of *loxP* (locus of X-over in P1) sites. Spatial expression of Cre is achieved using cell-specific *cre* promoters, whereas inducible systems can restrict expression temporally. The location and orientation of the *loxP* sites dictate whether Cre initiates deletion, inversion, or translocation of the “floxed” (flanked by *loxP*) locus. Typically, *loxP* sites are inserted a short distance apart on the same chromosome, in the same orientation, resulting in excision of the floxed segment and preventing successful production of a functional gene product (Fig. 1A). Alternatively, a floxed STOP codon may be inserted, whereby transcription of the downstream sequence, commonly a fluorescent reporter construct, only proceeds following Cre-mediated excision (Fig. 1A). Thus, Cre-lox permits intrinsic cellular labeling through cell-specific and, if required, temporally restricted, fluorescent protein expression. These manipulations require at least two Tg modifications, inserting Cre and *loxP* sites respectively, and are achieved by interbreeding strains carrying a single transgene. Therefore, this permits essentially any combination of Cre driver and floxed allele.

### “Conditional” Cre Expression

Cell-specific Cre expression is achieved using a cell-specific promoter element. In constitutive systems, promoter activation leads directly to Cre expression and recombination. However, constitutive systems do not permit a defined cohort of cells to be labeled at a specific point in time (temporal Cre expression), which negates their use in true fate-mapping experiments. Furthermore, constitutive Cre-driven ablation of developmentally important genes, even if cell specific, has the potential for embryonic lethality. As such, use of inducible Cre-lox systems, in which an exogenous stimulus is required to permit Cre-mediated recombination, has increased in recent years. This temporal regulation of Cre can be mediated through systems including Cre fusion proteins or the tetracycline (Tet) system.

The commonly used tamoxifen-inducible system employs a Cre fusion protein, CreER^T2^, containing a mutated ligand-binding domain of the estrogen receptor.^2^ This construct prevents Cre from entering the nucleus and driving recombination until exogenously administered tamoxifen binds to the fusion protein (Fig. 1B). Two principle versions of the Tet-inducible system exist: Tet-ON and Tet-OFF.^2^ Both rely on the insertion of *TetO* operator sequences upstream from a gene of interest and the separate incorporation of a tetracycline activator (TA) protein (tTA or rtTA) into the genome. Tet administration, usually as doxycycline, then either promotes (Tet-ON) or prevents (Tet-OFF) expression of the gene of interest. The Tet system’s main strength is the potential for reversible gene (in)activation. It can also be combined with Cre-lox, by either placing *cre* downstream of the *TetO* sequences or using Cre to switch on rtTA or tTA transcription.

Both tamoxifen and Tet systems require careful dose titration to maximize recombination efficiency while minimizing unwanted side effects. Low-level background recombination and poor recombination efficiency can be problematic. Tamoxifen may persist in the liver for 1-4 weeks after injection, and higher doses can cause nonspecific recombination.^3^ Either may confound accurate fate mapping by labeling additional cell types. As such, experimental design must be carefully considered, with use of appropriate controls crucial to confirm that observed effects are the result of the intended Cre-lox manipulation.

### Viral Vector-Mediated Regulation of Cre Expression

Viral vectors can also be employed to enable transgene expression in specific cell types in mice. Recombinant adeno-associated viruses are nonpathogenic helper-dependent parvoviruses with viral encoding proteins replaced by a gene or construct of interest, such as *cre*. This system has been utilized for elegant fate-mapping studies in the liver using a hepatocyte-targeted, adeno-associated virus containing Cre.^4^,^5^

### Flp-FRT

Analogous to Cre-lox, but of fungal rather than viral origin, Flp*-FRT* is an alternative system in which targeted genetic manipulation occurs through flippase (Flp)-mediated recombination of inserted flippase recognition target (FRT) sites.^2^ The recombination efficiency and thermostability of Flp have lagged behind that of Cre, although adoption of Flp variants, such as FLPe and FLPo, have greatly increased its utility.^2^,^6^ The system is already commonly used to remove antibiotic resistance genes during generation of Tg mouse strains. Although not yet widely adopted in the liver, use of Flp-*FRT* may well expand in coming years. Specifically, combination with Cre-lox allows independent or sequential gene manipulation, which may greatly aid modeling of cancer development in mice.^7^

### Reporter Systems

**Figure 2:**
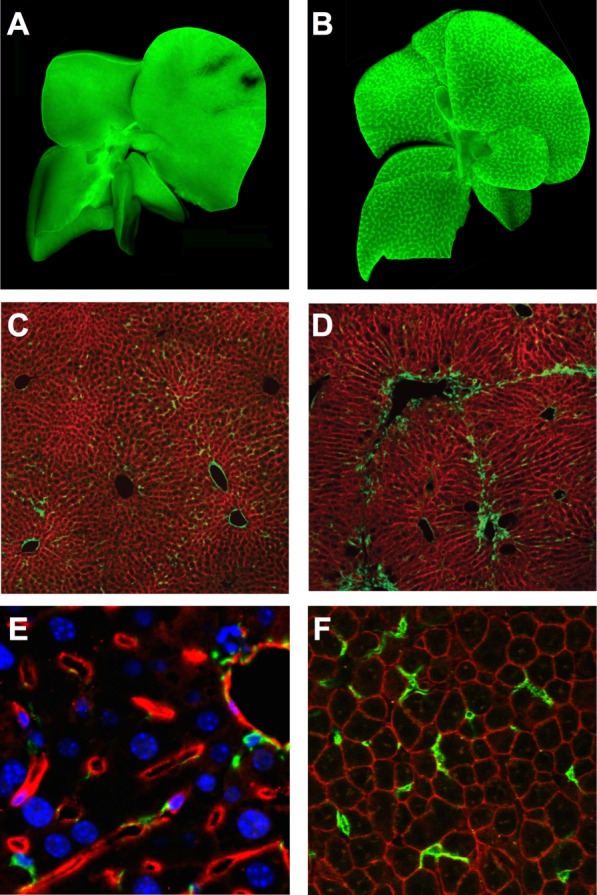
Labeling of hepatic NPCs with fluorescent reporters. (A and B) Lrat-Cre-driven ZsGreen labeling of qHSCs and aHSCs in whole livers from untreated (A) and CCl_4_-treated (B) mice. Adapted by permission from Macmillan Publishers Ltd: Nat Commun,^12^ © 2013. (C and D) Pdgfrb-Cre-driven membranous GFP labeling (green) of qHSCs and aHSCs in Pdgfrb-Cre;mTmG mice following olive oil (control, C) or chronic CCl_4_ (D) administration. Adapted by permission from Macmillan Publishers Ltd: Nat Med,^13^ © 2013. (E) Tamoxifen-induced Cdh5-PAC-CreER^T2^-driven TdTomato (red) labeling of LSECs. Nuclei (blue), α-SMA^+^ cells (green). Adapted from Supplementary Information to a previous work,^29^ by permission from the authors. (F) LysM-Cre-driven membranous GFP labeling (green) of macrophages in uninjured liver of LysM-Cre;mTmG mice (K.P.C. and N.C.H., unpublished data).

Cre-lox is an extremely powerful tool to label cells permanently and heritably, as required for fate-mapping studies. The ROSA26 locus is frequently used for the generation of “Rosa26R” reporter strains owing to its robust and ubiquitous expression.^8^ Common fluorescent reporter constructs (Fig. 2) include ZsGreen, TdTomato, membrane-targeted tdTomato membrane-targeted enhanced green fluorescent protein (mTmG), variants of enhanced green fluorescent protein (eGFP), and multicolor reporters, such as Confetti.^9^ Fluorescent constructs are constantly evolving to maximize fluorescence and fidelity and incorporate additional refinements, such as localization tags to allow cytosolic, membrane, and nuclear targeting.^9^ Fluorescent reporters also permit cell sorting without additional antibody staining and are of particular use when isolating cell types present at low frequency.

### Potential Cre Pitfalls

Off-target effects of Cre (“Cre toxicity”) have been reported and may occur through a variety of mechanisms, including insertion site activation or silencing, undocumented gene expression patterns, incomplete incorporation of driver construct regulatory elements, transgene silencing, and germline expression.^10^ Unintended Cre expression (“leakiness”) in nontargeted lineages or at inappropriate times may confound the conclusions of both gene deletion and fate-mapping experiments. Therefore, it is essential that the recombination efficiency (sensitivity) and expression pattern (specificity) for each Cre strain is carefully characterized and appropriate controls are selected.

## Targeting Hepatic NPCs

**Figure 3:**
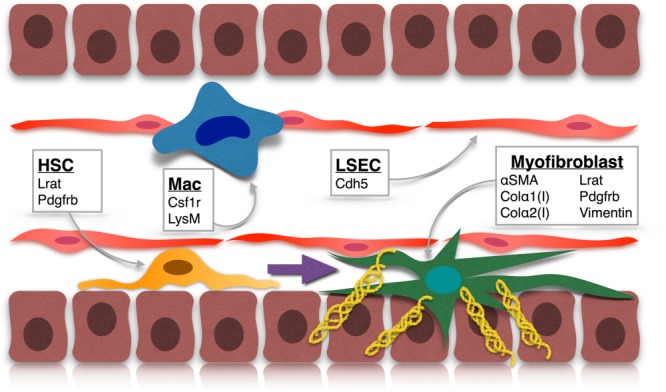
Tg approaches to targeting NPCs in the liver. qHSC: Lrat and Pdgfrb; Mac (macrophage): Csfr1 and LysM; LSEC: Cdh5; myofibroblast: α-SMA, Col-α1(I), Col-α2(I), Lrat, Pdgfrb, and vimentin.

Defining the role of NPCs in liver disease is vital to understand the pathobiology driving disease progression, particularly given the common pathway of inflammation to fibrosis to organ failure shared by most chronic liver diseases. The Cre drivers currently utilized to target hepatic NPCs are listed in Table 1, and the principal cell targets are illustrated in Fig. 3. Harnessing Tg mouse technology in models of human liver disease should allow for the identification of novel therapeutic targets. Given that there is now strong evidence that liver fibrosis is reversible,^11^ determining the means by which hepatic stellate cells (HSCs), myofibroblasts, macrophages, and liver sinusoidal endothelial cells (LSECs) contribute to progression and regression of liver fibrosis, as well as hepatic regeneration, is of great importance.

**Table 1:** Cre Drivers That May Be Used to Target Hepatic NPCs

Hepatic NPC Type	Cre Driver	Cre System	Other Relevant Cells That May Be Targeted	References
HSC/myofibroblast	Lrat	c	VSMC	(12)
	Pdgfrb	c	VSMC	(13)
	GFAP	c, i	Ductular cell, cholangiocyte	(12,14-17)
Myofibroblast	α-SMA	i	VSMC	(17,19)
	Colα1(I)	c		(16)
	Colα2(I)	c, i		(16)
	Vimentin	i	HSC, VSMC, portal fibroblast	(25)
LSEC	Cdh5	i		(29)
KC/macrophage	LysM	c	Granulocyte, DC	(34,36-38)
	Csf1r	i	Granulocyte, DC	(42,43)

Abbreviations: c, constitutive; i, inducible.

### Targeting HSCs and Myofibroblasts

Myofibroblasts are the principal profibrogenic cells in the liver, and there is now substantial *in vivo* evidence that HSCs, liver-specific pericytes residing in the space of Disse, are their major precursor.^12^ Thus, HSCs and liver myofibroblasts are of profound interest in the search for potent antifibrotic treatments.

### Lecithin-Retinol Acyltransferase

This HSC-targeting strategy capitalizes on their specialized role in retinoid storage, employing a Tg mouse in which Cre expression is driven by lecithin-retinol acyltransferase (Lrat), an enzyme with a key role in HSC lipid droplet formation.^12^ In two fluorescent reporter systems, Lrat-Cre labeled 99% of HSCs (defined by vitamin A fluorescence on flow cytometry). Confocal microscopy revealed strong colocalization with the HSC markers, desmin and platelet-derived growth factor receptor beta (PDGFRβ). Furthermore, Lrat protein expression was not identified in any other hepatic cell type. Only rare labeling of vascular smooth muscle cells (VSMCs) was observed (<1 per 200 HSCs). Thus, this strategy targets HSCs efficiently and with high specificity.

In models of toxic, cholestatic, and fatty liver disease, Lrat-Cre-driven reporter expression showed that 82%-96% of hepatic myofibroblasts, in part identified by concomitant expression of a collagen (Col)-GFP transgene (in which GFP is expressed under control of the Col-α1 (I) promoter/enhancer), derive from HSCs. However, HSCs did not function as epithelial progenitors. Combining Lrat-Cre with a Cre-inducible diphtheria toxin receptor resulted in marked HSC depletion and reduction in alpha-smooth muscle actin (α-SMA) and profibrogenic gene expression after CCl_4_ administration, again underlining the central role of HSCs in matrix deposition during liver fibrogenesis.

### Platelet-Derived Growth Factor Receptor Beta

Pdgfrb-Cre mice represent an alternative strategy to target HSCs and myofibroblasts.^13^ These mice express Cre under control of a *Pdgfrb* gene fragment and drive efficient recombination in both quiescent HSCs (qHSCs) and activated myofibroblasts with a high degree of specificity.^13^ A Pdgfrb-BAC-eGFP reporter confirmed PDGFRβ expression by qHSCs and offers an additional genetic strategy for fluorescent labeling of HSCs. The Pdgfrb-Cre strategy identified a key role for myofibroblast αv integrins in regulating fibrosis in several solid organs, including liver, lung, and kidney. Selective depletion of αv integrins from HSCs protected mice from CCl_4_-induced hepatic fibrosis, and αv-null HSCs had reduced ability to activate latent transforming growth factor beta. These findings were further validated using a small-molecule inhibitor to target αv integrins, which ameliorated fibrosis in both liver and lung.

### Glial Fibrillary Acidic Protein

A number of studies have used glial fibrillary acidic protein (GFAP)-Cre to target HSCs constitutively or inducibly.^14^–^17^ However, it has been suggested that GFAP-Cre does not target HSCs with high specificity. Using a GFAP-Cre mouse (in which the human GFAP promoter controls Cre expression) crossed to a GFP reporter, GFAP, Cre-recombinase, and GFP reporter protein were detected in both HSCs and ductular cells of the biliary system.^14^ GFAP ductular expression was also confirmed in human and rat liver. Subsequently, GFAP-Cre reporters have demonstrated labeling of bile ducts and cytokeratin 19–expressing cholangiocytes, rather than desmin^+^ HSCs or collagen-producing myofibroblasts.^12^ Thus, it appears that GFAP-Cre does not target HSCs exclusively and its overall utility in murine studies of liver disease has still to be fully clarified.

### α-SMA

Myofibroblasts are classically identified by α-SMA expression.^18^ An inducible Cre linked to α-SMA (α-SMA-Cre-ER^T2^) has been employed to target hepatic myofibroblasts.^17^,^19^ High recombination rates (57%-81%) were achieved when using α-SMA-Cre-ER^T2^ to abrogate smoothened expression from α-SMA^+^ liver cells and loss of smoothened disrupted Hedgehog signaling and reduced fibrosis after bile duct ligation (BDL). Myofibroblast accumulation and hepatocyte proliferation were also inhibited following BDL or partial hepatectomy (PH). Interestingly, when α-SMA-Cre-ER^T2^ mice were crossed with a yellow fluorescent protein (YFP) reporter, YFP expression was reported in 8%-34% of hepatocytes following PH or BDL. This finding raised the possibility that the α-SMA^+^ myofibroblast population includes hepatocyte precursors, contrary to current dogma and subsequently challenged by alternative fate-mapping approaches.^12^,^20^,^21^

### Collagen

Activated HSCs (aHSCs) are the major source of collagen within the liver,^12^,^22^,^23^ and several Tg mouse strains exist in which collagen-expressing cells have been targeted.^16^ In a Col-GFP Tg mouse, following 2 months of liver injury with CCl_4_, 92.6% of all GFP^+^ cells in the nonparenchymal fraction were α-SMA^+^, demonstrating good specificity for myofibroblasts. Further characterization of these GFP^+^ cells suggested that HSCs (vitamin A^+^ and desmin^+^) are the major source (92%) of myofibroblasts in this model of liver injury. Col-GFP mice have also enabled phenotypic analysis of the GFP^+^ myofibroblast population following BDL.^24^ In contrast to CCl_4_ injury, vitamin A^−^ cells initially (days 5 and 17 post-BDL) formed the majority of myofibroblasts following cholestatic liver injury. Additional characterization defined a Thy1^+^, elastin^+^, and desmin^−^ population that was suggested to comprise portal fibroblasts.

Col-α1(I) and Col-α2(I) form the triple helix of collagen type I. In mice containing either the constitutive Col-α1(I)-Cre or Col-α2(I)-Cre transgenes, each crossed with a YFP reporter and following 2 months of CCl_4_, >92% of α-SMA^+^ cells and >94% of desmin^+^ cells expressed YFP reporter, providing evidence that recombination occurs with high efficiency in myofibroblasts/aHSCs.^16^ Furthermore, when a tamoxifen-inducible Col-α2(I)-CreER was crossed with a mTmG reporter, ∼35% of desmin^+^ HSCs were labeled by 7 daily doses of tamoxifen administered after 7 weeks of CCl_4_.

These three targeting-reporter strategies revealed that hepatic myofibroblasts can revert to an inactive HSC phenotype during regression of CCl_4_-induced fibrosis.^16^ In the Col-α2(I)-CreER targeting strategy, 14% of desmin^+^ HSCs were GFP^+^ 1 month after recovery, almost half the number labeled at peak fibrosis. Similar results were observed in a model of alcohol-induced liver fibrosis. Notably, Col-GFP mice receiving a second course of CCl_4_, after complete recovery from the first, developed more-severe fibrosis than previously untreated littermate controls receiving CCl_4_ for the first time.

### Vimentin

Vimentin is a cytoskeletal filament protein strongly expressed in mesenchymal cells, particularly myofibroblasts. A tamoxifen-inducible Cre under control of the vimentin promoter has been employed to track activation and reversion of HSCs and myofibroblasts following cessation of liver injury.^25^ Minimal reporter expression was detected in uninjured liver, suggesting that this strategy is unsuitable for targeting qHSCs. Following chronic CCl_4_ injury, ∼25% of HSCs were labeled, with all labeled cells desmin^+^.

### Targeting LSECs

LSECs comprise a unique endothelial subpopulation lining hepatic sinusoids. They differ both morphologically and functionally from the endothelia of other organs, with numerous fenestrae, no basement membrane, and a diverse range of roles.^26^ Although there are currently no LSEC-specific Cre drivers, a general endothelial-targeted Cre driver has recently been successfully employed to target LSECs.

### Vascular Endothelial Cadherin

Vascular endothelial cadherin is a cell-cell glycoprotein coded for by the *cdh5* gene and is the major cadherin expressed by LSECs.^27^ Its key functions are endothelial cohesion and intercellular junction organization. A tamoxifen-inducible Cdh5-PAC-CreER^T2^ strain, initially utilized in studies of angiogenesis,^28^ successfully targets LSECs.^29^ Following tamoxifen, TdTomato reporter expression was widespread in endothelial cells, but did not label desmin^+^, PDGFRβ^+^, or α-SMA^+^ cells. The Cdh5-PAC-CreER^T2^ strategy was then employed to knock down LSEC expression of C-X-C chemokine receptor (CXCR)4, CXCR7, or fibroblast growth factor receptor 1 (FGFR1). LSEC-specific *Cxcr7* knockdown significantly reduced hepatocyte proliferation after acute liver injury (single-dose CCl_4_) and, following chronic injury (BDL or chronic CCl_4_), impaired regeneration and promoted fibrosis. Conversely, loss of CXCR4 or FGFR1 in LSECs limited the profibrotic changes occurring after BDL, demonstrating that FGFR1-mediated CXCR4 up-regulation can counterbalance the LSEC proregenerative response and promote fibrosis. Thus, LSECs, through CXCR7 and CXCR4, play a key role in liver injury and repair.

### Targeting Macrophages in the Liver

Liver injury stimulates both activation of Kupffer cells (KCs), the liver-resident macrophages, and infiltration of circulating macrophages. Macrophage subpopulations are critical for both fibrogenesis and its resolution,^30^ and it is therefore essential to dissect the underlying mechanisms regulating these divergent responses to injury. Currently, the greatest challenges in Tg targeting of liver macrophages are unresolved, namely, distinguishing macrophages from other cells of the myeloid lineage and the ability to target liver-resident macrophages separately from recruited macrophage populations.^31^ Despite these limitations, the Tg approaches outlined below have proved useful tools for targeting myeloid cell populations within the liver.

### M Lysozyme

In mice, M lysozome (LysM) is specifically expressed in myeloid cells and up-regulated following macrophage activation.^32^ In the LysM-Cre mouse, recombination rates of 83%-95% were achieved in peritoneal macrophages positive for the classical macrophage marker, F4/80.^33^ However, recombination was also observed in up to 99% of thioglycollate-elicited peritoneal neutrophils and a minority of dendritic cells (DCs). Recently, it was shown that variable LysM expression by macrophage subsets can result in differential recombination, with immature macrophages showing less recombination than mature, tissue-resident macrophages.^34^ This work also elegantly demonstrated the differing roles of macrophage subpopulations during schistosomiasis-induced hepatic fibrosis.

LysM-Cre, combined with a GFP reporter, has also been employed to track loss of KC membrane integrity following Ad5 adenovirus infection,^35^ and in further gene deletion studies.^36^–^38^ Using LysM-Cre to knock down peroxisome proliferator-activator receptor gamma (PPAR-γ) was shown to reduce macrophage expression of both PPAR-γ isoforms to <25% of baseline and, in both acute and chronic CCl_4_-induced liver injury, these mice demonstrated increased liver injury and fibrosis.^38^

### Colony-Stimulating Factor 1 Receptor

The *c-fms* gene encodes colony-stimulating factor 1 receptor (CSF1R), expression of which is restricted to cells of the myeloid lineage (and placental trophoblasts).^39^ The “MacGreen” knock-in mouse combines the proximal promoter and first intron of the *c-fms* gene with the coding sequence for eGFP, labeling macrophages throughout the mouse.^39^ In liver, eGFP^+^ cell location and morphology was similar to that of F4/80^+^ cells, although simultaneous costaining was not performed in the initial characterization. Although transgene expression initially appeared restricted to monocytes and macrophages, expression in granulocytes^40^ and DCs^41^ was subsequently confirmed.

The *Csf1r* promoter has also been employed to target a tamoxifen-inducible Cre (Csf1r-Mer-iCre-Mer) to myeloid cells, and its initial characterization demonstrated a reduction in peripheral blood monocyte vascular endothelial growth factor A expression to 16% of baseline.^42^ No reduction in expression was observed in other circulating immune cells, including granulocytes. A key advantage of the Csf1r-Mer-iCre-Mer mouse is that it permits both cell-specific and temporal control of Cre-mediated recombination. This has enabled pulse labeling and lineage tracing of yolk sac macrophages in developing embryos, distinguishing them from macrophages derived from hematopoietic stem cells later in gestation.^43^ Although a relatively small proportion (0.3%-6.3%) of tissue macrophages were labeled by this strategy, a similar approach could allow macrophage targeting at specific time points during liver injury and regeneration and may therefore be useful in fate-mapping studies of KCs.

## Challenges and Future Directions

Tg mouse technology continues to evolve rapidly, advancing our understanding of the cellular and molecular mechanisms regulating hepatic injury, wound healing, and regeneration. Novel Cre drivers, with improved specificity and efficiency, and increasing adoption of inducible Cre strategies will enable high-fidelity interrogation of the precise role of individual cell lineages during the development and resolution of liver disease. Fluorescent reporter systems also offer exciting potential for further exploitation; in addition to fluorescent cell sorting, combining fluorescent reporters with techniques such as laser capture microdissection, single-cell real-time polymerase chain reaction and even single-cell RNA sequencing, offers enormous opportunities.

However, it is critically important that, as Tg technology advances, a rigorous approach to its utilization is applied in order to ensure that robust, high-quality data are generated. Excellent catalogs of available Tg strains are already in existence, but it is essential that researchers developing new mouse strains, or utilizing existing strains in novel applications, characterize them fully with regard to targeting efficiency and specificity.

Through an increasingly finessed approach to genetic manipulation, we are gaining an ever more detailed picture of the cellular and subcellular processes that regulate hepatic pathology. Harnessing Tg technology in the liver will help identify rational antifibrotic and proregenerative targets, facilitating efficient translation of scientific discoveries into potent therapies for patients.

## Notes

Author names in bold designate shared co-first authorship.

## Abbreviations

aHSCs: activated HSCs
α-SMA: alpha-smooth muscle actin
BDL: bile duct ligation
Col: collagen
Cre: cyclization recombinase
CSF1R: colony-stimulating factor 1 receptor
CXCR: C-X-C chemokine receptor
DCs: dendritic cells
eGFP: enhanced green fluorescent protein
FGFR1: fibroblast growth factor receptor 1
Flp: flippase
FRT: flippase recognition target
GFAP: glial fibrillary acidic protein
GFP: green fluorescent protein
HSC: hepatic stellate cell
KC: Kupffer cell
loxP: locus of X-over in P1
Lrat: lecithin-retinol acyltransferase
LSEC: liver sinusoidal endothelial cell
LysM: M lysozyme
mTmG: membrane-targeted tdTomato membrane-targeted eGFP
NPCs: nonparenchymal cells
PDGFRβ: platelet-derived growth factor receptor beta
PH: partial hepatectomy
PPAR-γ: peroxisome proliferator-activator receptor gamma
qHSCs: quiescent HSCs
TA: tetracycline activator
Tet: tetracycline
Tg: transgenic
VSMCs: vascular smooth muscle cells
YFP: yellow fluorescent protein
